# Assessment of Surface Integrity in Precision Electrical Discharge Machining of HSS EN HS6-5-2C

**DOI:** 10.3390/mi15121469

**Published:** 2024-12-01

**Authors:** Ľuboslav Straka, Ján Piteľ, Ivan Čorný

**Affiliations:** 1 Department of Automobile and Manufacturing Technologies, Faculty of Manufacturing Technologies with the Seat in Presov, Technical University of Kosice, Sturova 31, 080 01 Presov, Slovakia; 2 Department of Industrial Engineering and Informatics, Faculty of Manufacturing Technologies with the Seat in Presov, Technical University of Kosice, Bayerova 1, 080 01 Presov, Slovakia; jan.pitel@tuke.sk; 3 Department of Process Engineering, Faculty of Manufacturing Technologies with the Seat in Presov, Technical University of Kosice, Sturova 31, 080 01 Presov, Slovakia; ivan.corny@tuke.sk

**Keywords:** High-Speed Steel (HSS), quality, Surface Crack Density (SCD), Wire Electrical Discharge Machining (WEDM)

## Abstract

The integrity of the machined surface in precision wire electrical discharge machining (WEDM) of electrically conductive materials is one of the most important quality indicators. The integrity parameters of the machined surface are primarily monitored in terms of micro and macro geometry parameters. This paper presents the results obtained as a part of experimental research aimed at evaluating surface crack density (SCD) when machining EN HS6-5-2C using WEDM technology. The aim was to find a combination of main technological parameters (MTP) in order to minimize the qualitative indicators SCD and Ra of the eroded surface. The results of experimental research within the framework of the evaluation of SCD and Ra indicators were processed using the Taguchi method. The integrity of the eroded surface was examined by scanning digital microscope (SDM) after application of full and multiple offset cuts with an AC Brass LP 1000 brass wire electrode. Based on the experimental measurements performed, significant facts were discovered. It was found that the largest surface integrity defects are present after the application of full cuts and the first two offset cuts. At the same time, it was found that lower values of the SCD parameter in WEDM of EN HS6-5-2C steel were recorded at thicknesses above 130.0 mm. The SCD parameter was also confronted with the Ra parameter, and it was found that they are significantly influenced by MTP. The higher value of the peak current *I* (19 A) and the longer duration of the discharge *t_on_* (32 μs) result in an increase in the value of the SCD parameter from 0.005 μm·μm^−2^ to 0.0256 μm·μm^−2^. The resulting solutions are mathematical regression models (MRM), which allow the prediction of both monitored qualitative indicators with respect to their minimization.

## 1. Introduction

One of the progressively developing machining technologies today is wire electrical discharge machining (WEDM). This is a technology that enables contactless machining of very hard materials. However, this technology also has its limits. One of them is the minimum electrical conductivity of the processed material at the level of 0.01 S·cm^−1^ [1,2]. With WEDM technology it is possible to machine intricate and complex shapes with high dimensional and geometric precision, which is practically impossible to achieve with other technologies. Typical example are internal sharp edges or very small internal rounding [3,4,5]. This machining technology has proven to be a valuable tool in machining high-strength and hard materials for the automotive and aerospace industries, as well as for other industries. Although high quality of the machined surface is achieved with WEDM, in certain specific cases the most demanding requirements for its quality indicators are not always met. In addition to the eroded area problems associated with deficiencies in geometric accuracy, no less serious are the problems associated with the integrity of the surface eroded layers. These are defects that are accompanied by the formation of cracks due to local heating during electrical discharge machining with subsequent rapid cooling of the surface due to the action of a dielectric liquid.

Several researchers have dealt with the issue of surface integrity assessment after WEDM in the past. Welling and Sharma [6,7] dealt with the surface integrity of Inconel 718 machined by WEDM technology. As part of the performed experimental research, they analysed the correlations between the surface integrity and the behavior of HCF on the bending fatigue test bed. Kumar et al. [8] performed an analysis of the surface integrity of the material l–MMC with 10% SiCp after EDM. They investigated the effect of input machining parameters, including discharge current, pulse on-time and gap voltage, on output responses, such as electrode wear, material removal rate, surface roughness, electrode temperature, workpiece temperature, microstructure, microhardness, SEM and EDX analysis. They found that discharge current, pulse on time and gap voltage have a significant effect on the mentioned responses. Evaluation of surface morphology, surface roughness, elemental composition, microhardness and residual stress of the machined surface by different WEDM strategies was carried out by Mandal et al. [9]. As part of the experimental findings, they proposed to improve the surface integrity of the machined surface after WEDM through its grinding and etching. Thrinadh et al. [10] studied the surface morphology along with topographic features including average roughness, crack density, remelted layer thickness, material immigration, residual stress and microhardness of Ti–6Al–4V material machined by WEDM technology. The obtained results were analysed with respect to the gap width, material removal rate and wire wear. White layer thickness and crack density in WEDM machining of YG15 were studied by Zhang et al. [11]. As part of the conducted experimental research, they investigated the influence of process parameters on the integrity of the surface. The results of their research showed that pulse duration and discharge current have a significant effect on surface roughness (SR), white layer thickness (WLT) and surface crack density (SCD). Antar et al. [12] investigated the effects of surface integrity of EDM machined AISI surface on fatigue performance. They found out that the samples showed a slightly lower fatigue performance, as a result of surface microcracks. The influence of the main parameters of the electro-erosion process and the powder concentration in PMEDM with tungsten carbide powder on the main responses in the machining process of steel SKD61) were investigated by Tao Le [13]. As part of the research, he analysed the effect of peak current and pulse on-time on the surface integrity of the remelted layer. Their upward trend was manifested with higher values for the parameters. The study of the input parameters of the electro-erosion process and the effect of the dielectric to improve the topography of the machined AISI 1045 material was dealt with by Rahimi et al. [14]. They found that using deionized water as a dielectric reduced microcracks and the thickness of the heat-affected zone (HAZ). Zeilmann et al. [15] presented a study of the surface integrity of AISI H13 steel, machined by the EDM process using constant parameters and a copper electrode with different cavity depths and pulse times. They examined texture, crack density, surface roughness, and affected layer. They found that the depth and concentration of cracks increased with increasing pulse on-time and cavity depth. Mascaraque-Ramirez et al. [16] used three different methods of experimental analysis of the surface quality of AISI 316 stainless steel in terms of its morphology and integrity as part of an experimental investigation. They found that the crater extent is the best indicator to characterize the quality of the surface in terms of its integrity during electric discharge machining, as it shows a clearer tendency as a function of current intensity and penetration depth.

Based on the analysis of the current state of HSS surface integrity after WEDM, it can be concluded that only a few researchers have contributed to the evaluation of the extent of microcracks depending on the machining method and the roughness of the eroded surface. At the same time, there is no research aimed at assessing the presence of disintegrity of the eroded surface in its entire cross-section depending on the thickness of the machined material. At the same time, surface integrity, together with dimensional accuracy and roughness, is one of the important qualitative indicators of the machined surface [17,18,19,20,21]. Therefore, the research was aimed at identifying selected parameters of the integrity of the machined surface of HSS EN HS6-5-2C after WEDM with a brass wire electrode. The research was conducted on HSS samples, which can be characterized as a tool steel with a high content of alloying elements. These alloying elements significantly improve its cutting properties. The main alloying element of HSS is W (wt. 5 to 20%), which forms a tungsten carbide compound with high hardness and resistance to abrasion with C in the steel structure. Another alloying element is Cr (wt. approx. 4%), which improves the hardenability of HSS and, in combination with V (wt. 1 to 4%), the resistance to tempering. An important alloying element is also Co (wt. 5 to 10%) which, in combination with the carbon in the steel, will form the number of carbides necessary for HSS to be hardenable and sufficiently hard at the same time. In addition to good cutting properties, HSS is characterized by favourable mechanical properties, such as hardness, strength, and toughness, even under high mechanical and thermal loads. Therefore, these materials are successfully applied in the production of cutting tools. However, their specific properties result in extensive disintegration of surface layers when an inappropriately selected combination of MTPs is used in the electro-erosion process. Furthermore, it is assumed that the surface disintegrity during machining of given steels is not consistent across the entire cross-section of the machined surface. Therefore, within the framework of our experimental research, the integrity was evaluated across the entire cross-section of the eroded surface with different heights of machined material using experimental and numerical methods. The research was aimed at evaluating surface integrity parameters for different experimental conditions with the application of the Taguchi method [22,23,24,25,26]. Main technological parameters (MTP) were considered as input variable parameters. By applying the experimental technique, parameters of surface roughness Ra and its integrity, such as SCD, were identified in the entire cross-section of the machined surface during individual WEDM operations [27,28,29,30,31,32]. At the same time, 2D graphical dependence of the output monitored indicator SCD and Ra of the eroded surface with respect to the height of the machined surface *H* and the input variables MTP of the electro-erosion process were constructed. Subsequently, based on the performed numerical estimation, mathematical regression models (MRM) were determined, including 3D visualization for the prediction of SCD in a wider dimensional spectrum of height *H* of high-speed steel EN HS6-5-2C machined by WEDM technology. Finally, the findings were compared with the results achieved by other researchers in the given field of research.

## 2. Identification of the Integrity of the Eroded Surface After WEDM

**Figure 1:**
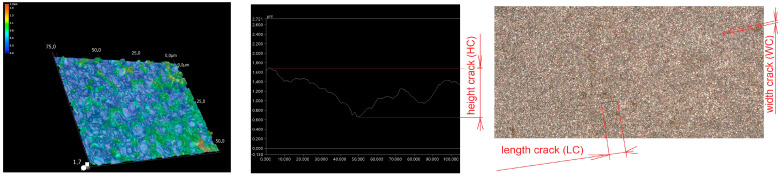
Parameters of a crack on an eroded surface.

Based on the analysis of the current situation in the field of defects of the machined surface, it was found that the generation of thermal stress with a magnitude greater than the fracture strength of the machined material leads to the formation of surface cracks [33]. They are primarily involved in its disintegrity [34,35,36,37,38]. This is also the case with WEDM technology. The electro-erosion process causes rapid local heating of the machined material, as a result of which melting and evaporation of the contact particles of the material occurs, followed by rapid cooling of the base material through the dielectric liquid under the simultaneous periodic action of electrical discharges. The result is damage to the eroded surface in the form of cracks [39]. Cracks on the machined surface are a serious problem in terms of the material’s resistance to fatigue, tensile stress, but also because of the increased susceptibility to surface corrosion [40,41,42,43,44,45]. These surface integrity defects can be quantified in terms of width, length or depth, or according to the number of cracks. Figure 1 shows the basic parameters of the cracks that occur on the eroded surface.

One of the important indicators of the number of cracks on the eroded surface is the SCD. This indicator shows the share of the total length cracks LC (µm) per unit of the assessed area A (µm^2^) according to the Formula (1):

(1) $$\mathrm{SCD}=\frac{\mathrm{LC}}{A}$$

**Figure 2:**
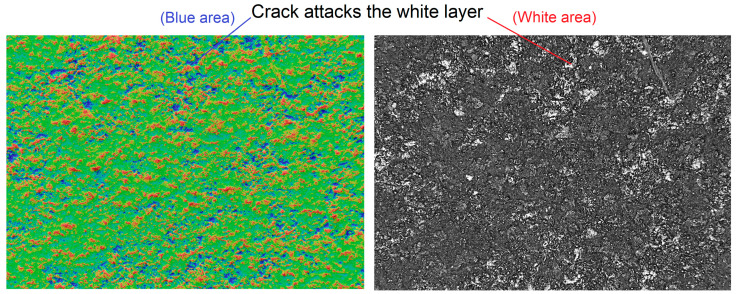
A crack passing through the white layer of the machined surface into the base material.

As already mentioned, high thermal stresses occur in the subsurface layers of the eroded material, which generally exceed the fracture strength of the base material [46,47,48,49]. In addition, the eroded surface is exposed to chemical contamination, especially by C. Its contamination occurs due to the influence of the wire tool electrode interacting with the dielectric liquid. At the same time, from a metallurgical point of view, C can be considered the most significant contamination of the eroded surface. At the same time, the saturation of the eroded surface by C increases the proportion of the white layer and its fragility, while the high fragility of the white layer on the eroded surface gives a favourable condition for the formation and development of cracks. Figure 2 shows a crack that passes through the white layer of the machined surface into the base material. This is a crack that emerged as a result of intense heating followed by rapid cooling of the surface during WEDM.

The integrity of the surface after WEDM needs to be evaluated, not only based on the amount, but also the extent of these cracks [50,51,52]. Cracks that penetrate through cross sections are particularly dangerous. These are cracks that are present on the eroded surface and spread towards the interior of the base material. They are mainly caused by the shrinkage of the material during solidification as a result of the rapid cooling of the molten metal by the dielectric liquid.

## 3. Materials and Methods

### 3.1. Description of the Material of the Samples, the Cutting Tool and the Machine Used

Within the research, HSS EN HS6-5-2C (W.-Nr. 1.3343) was used to make the experimental samples. This is a high-speed steel alloyed with W and Mo, which is characterized by a high tensile strength of up to 915 MPa at high temperatures, good toughness and abrasion resistance. It is difficult to form when hot, with reduced machinability in the soft annealed state. It is susceptible to decarburization during heat treatment. It is used for the production of high-performance tools with a good hardness/toughness ratio, such as milling cutters, drills, reamers, tapers, turning, shaping and planing knives, segments for metal circular saws, forming and shearing tools. Its chemical composition is (wt%) C 0.9; Cr 4.2; Mo 4.9; V 1.9 W; 6.4; Si max 0.45; Mn max 0.4; P max 0.03 and S max 0.03.

The parameters of selected physical and mechanical properties of the high-speed steel EN HS6-5-2C used are listed in Table 1.

**Figure 3:**
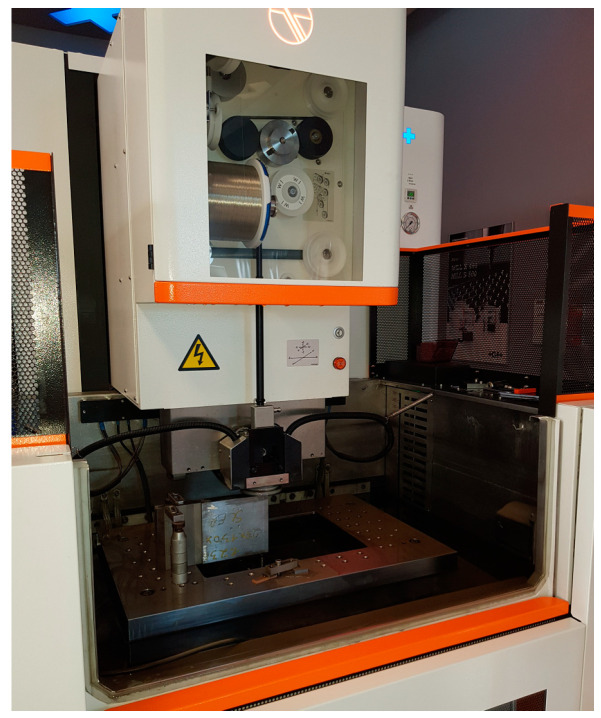
Electrical discharge device AgieCharmilles CUT E 350.

Experimental samples were made by WEDM technology on an AgieCharmilles CUT E 350 EDM machine (GF Machining Solutions–Headquarters, Biel, Switzerland) (Figure 3). This is a device of the Swiss manufacturer GF with a modern Intelligent Power Generator (IPG), which enables high cutting speed, high cutting accuracy and low roughness of the eroded surface. Thanks to UniQua’s intelligent and intuitive interface, which offers flexibility, it is possible to relatively efficiently produce various cutting tools, but also, for example, punches, moulds, etc. In addition, it has powerful tools for performing a fast and safe electro-erosion process. The main technical parameters of the electrical discharge device AgieCharmilles CUT E 350 are listed in Table 2.

The EN HS6-5-2C high-speed steel samples were machined using a wire tool electrode marked AC Brass LP 1000. The used brass wire tool electrode ∅ 0.25 mm is suitable for various WEDM operations, such as roughing, finishing, but also high-precision machining. The stability of the electro-erosion process in the case of the application of the given wire tool electrode is supported by the material composition of Cu/Zn in the ratio of 63:37%. The samples were made in the presence of a liquid dielectric based on deionized water, which meets all performance and safety requirements. Dielectric liquid was treated with a demineralization resin to the level of electrical conductivity of <10 μS·cm^−1^.

**Figure 4:**
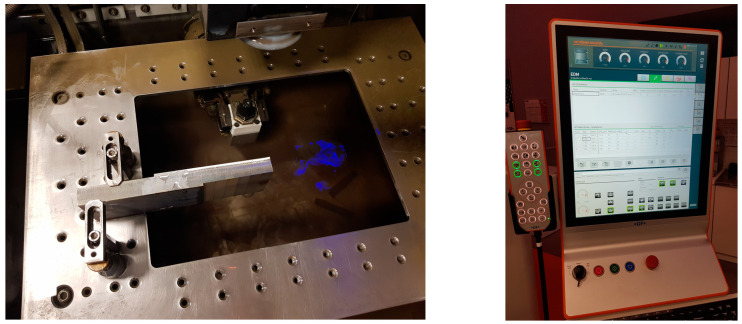
Production of experimental samples from high-speed steel HSS EN HS6-5-2C by WEDM technology.

Figure 4 shows the process of manufacturing experimental samples from high-speed cutting cell HSS EN HS6-5-2C by the electrical discharge equipment AgieCharmilles CUT E 350 with a wire tool electrode AC Brass LP 1000.

### 3.2. Method Design and Preparation of the Experiment

For well-organized experimental research in the field of surface integrity after WEDM, it is very important to design a suitable and efficient set of experiments to collect the required data [53]. The obtained data are thus intended to be used for the analysis of the dependent output variable from the input non-dependent parameters of the electro-erosion process at different levels. The goal is also to perform a quantitative formulation of the answers that will provide the desired information regarding the output of the experimental research. In doing so, the basic condition must be met that the outputs are realistically measurable. The input variable parameters of the electro-erosion process are defined within the design of the experiment as factors with different levels that can be used and maintained during the experiments with the required accuracy [54]. Subsequently, the individual effect of the input independent variable parameters of the electro-erosion process is assessed, as well as their interactive effect on the output dependent parameters. In doing so, it is possible to identify significant input independent process variables that influence the output dependent parameters of the electro-erosion process [55,56,57,58]. Through the performed analysis, it is also possible to identify the optimal settings of the input independent parameters of the electro-erosion process in order to achieve the required level of the output monitored indicator of the process. A suitable design of the experiment plan can also significantly influence the number of necessary experiments while, through a well-designed experimental plan, their number can be significantly reduced. Therefore, the design of the experiment plan is the first and most important step in conducting experimental research. Based on the analysis of several suitable methods, applicable in the design of the plan of experiments for making samples using WEDM technology, the Taguchi method was chosen. It is a method that can be a very effective tool in investigating the output response influenced by several input independent variables [59,60,61,62]. At the same time, it can also focus on minimizing the influence of the causes of variations with relatively little sensitivity to uncontrollable factors [63]. In addition, the advantage of applying this method compared to traditional fully factorial designs of experiments is that it allows a significant reduction in the size of the set of experiments and thus speeds up the experimental process. This is a huge advantage, especially in the electrical discharge machining process, which is particularly time-consuming to produce experimental samples [64]. As part of the design of the experiment, a 4-factor analysis was applied at 4 levels of input-dependent MTP settings of the electro-erosion process, namely peak current *I*, discharge voltage *U*, pulse on-time *t_on_* and pulse off-time *t_off_*. For each thickness of the machined material, 16 experimental samples were made.

Within the experiment, material thicknesses of 30.0 mm, 80.0 mm and 130.0 mm were evaluated, while a total of 48 experimental samples were made. The setting of the input MTPs of the electro-erosion process was implemented at four levels Very Low/Low/Middle/High. For *I* on the level 2/8/14/19A, for *t_on_* on the level 8/16/24/32 μs, for *t_off_* on the level 1/7/14/20 μs and for *U* on the level 60/70/80/90 V. Recorded results of experimental measurements of SCD during WEDM of high-speed steel EN HS6-5-2C with brass wire electrode AC Brass LP 1000 for thicknesses of the processed material 30.0 mm, 80.0 mm and 130.0 mm are shown in Table 3.

Experimental samples with MTP settings at the level of L13 to L16 were made when applying a full cut, at the level of L9 to L12 when applying the first offset cut, at the level of L5 to L8 when applying the second offset cut, and at the level of L1 to L4 during the third offset cut. From the results of the experimental measurements listed in Table 3, it can be observed that the value of the SCD integrity indicator of the machined surface of high-speed steel EN HS6-5-2C with material thickness *H* = 30.0 to 130.0 mm using WEDM technology was in the range from 0.0041 μm·μm^−2^ to 0.0298 μm·μm^−2^.

At the same time, the lowest value of the SCD indicator was recorded after the third offset cut when machining material with a material thickness of 130.0 mm and setting the MTP at the L1 level (*I* = 2 A, *t_on_* = 8 μs, *t_off_* = 1 μs a *U* = 60 V). Conversely, the highest value of the SCD indicator was recorded after a full cut when machining material with a material thickness of 30.0 mm and setting the MTP at the L16 level (*I* = 19 A, *t_on_* = 32 μs, *t_off_* = 1 μs a *U* = 80 V). The recorded results of experimental measurements also showed that lower values of the SCD indicator at identical MTP settings were recorded when machining materials with a thickness of *H* = 130.0 mm, on the contrary, higher values were recorded when machining materials with a thickness of *H* = 30.0 mm. At the same time, this difference was at the level of approx. 30%. At the same time, it can be observed that lower SCD values were recorded with multiple offset cuts. It follows that, in addition to MTP, the value of the SCD eroded surface integrity indicator is also affected by the thickness of the machined material *H*, while its value decreases as the thickness of the machined material increases. With the identical MTP setting, the average value of the index of integrity of the eroded surface SCD_av_ during WEDM of high-speed steel EN HS6-5-2C with a brass wire electrode in the range of material thicknesses from 30.0 mm to 130.0 mm ranges from 0.0050 μm·μm^−2^ to 0.0256 μm·μm^−2^.

### 3.3. Assessment of the Influence of Selected MTPs on the Integrity of the SCD Surface

Based on the performed preliminary analysis of the current state in the area of the influence of MTP on SCD in WEDM of high-speed steels, several contradictory statements were found. For example, Wang et al. [65] claim that the minimization of SCD is positively influenced by a high value of the discharge current and the pulse off-time and at the same time a low value of the pulse on-time. However, Shather et al. [66] also claim that the minimization of SCD is positively influenced by a low value of the discharge current and a high value of the pulse on-time and the pulse off-time. Since there are conflicting statements in several sources regarding the dependence of SCD on MTP during WEDM, it was necessary to perform a targeted analysis of the influence of selected input factors during EDM of high-speed steel EN HS6-5-2C with a brass wire tool electrode AC Brass LP 1000 on the output dependent indicator of the machined surface. The analysis was performed by the Taguchi method.

**Figure 5:**
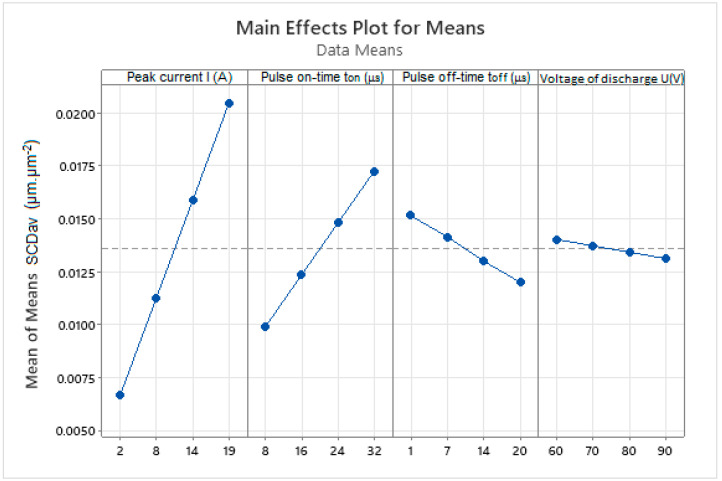
Analysis of the influence of selected MTP on the surface integrity parameter SCD_av_ during WEDM of high-speed steel EN HS6-5-2C.

Based on the average values of the output dependent indicator of the integrity of the eroded surface SCD_av_ recorded in Table 3, a 4-factor analysis of the influence of selected MTPs during WEDM of high-speed steel EN HS6-5-2C with brass wire electrode AC Brass LP 1000 was performed using the Taguchi method. The results of the performed 4-factor analysis of the influence of MTP on the output indicator of the integrity SCD_av_ of the machined surface at 4-levels of their settings are shown in the diagrams in Figure 5.

Based on the analysis of the influence of selected MTP on the surface integrity parameter SCD_av_ during WEDM of high-speed steel EN HS6-5-2C, several facts were found. First of all, it was found that parameter *I* has the greatest influence on the surface integrity parameter SCD_av_, followed by *t_on_*. *U* has the smallest influence of the assessed parameters. At the same time, it was found that the parameters *U* and *t_off_* have the opposite influence on the SCD_av_ than the parameters *I* and *t_on_*. This means that increasing the values of the parameters *U* and *t_off_* leads to an improvement in the quality of the machined surface in terms of its integrity. On the contrary, as the values of the parameters *I* and *t_on_* increase, the quality of the machined surface deteriorates in terms of its integrity, which is reflected in the increase in the SCD_av_ indicator value.

**Figure 6:**
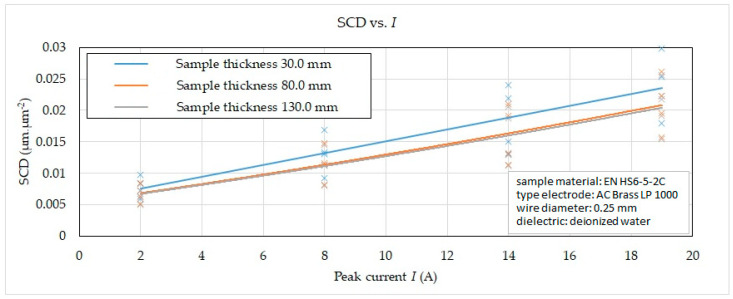
Effect of *I* and *H* on SCD in different WEDM methods.

**Figure 7:**
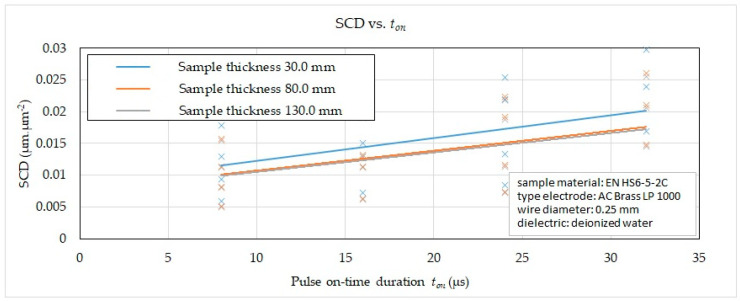
Effect of *t_on_* and *H* on SCD in different WEDM methods.

**Figure 8:**
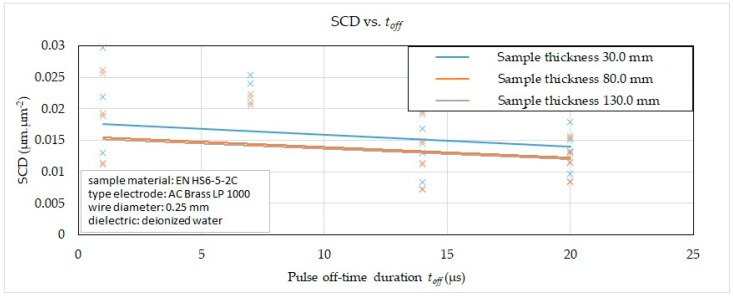
Effect of *t_off_* and *H* on SCD in different WEDM methods.

**Figure 9:**
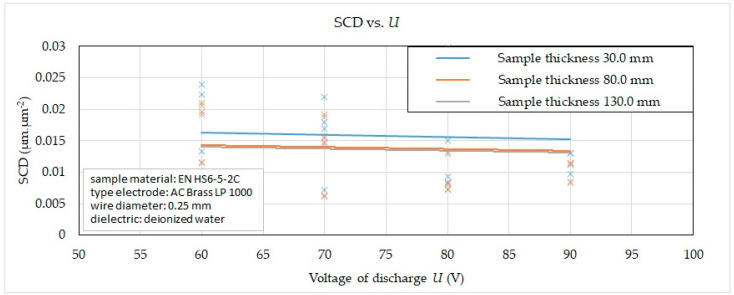
Effect of *U* and *H* on SCD in different WEDM methods.

Subsequently, on the basis of the recorded data, 2D graphic dependencies were constructed (Figure 6, Figure 7, Figure 8 and Figure 9) describing the influence of individual input independent MTP on the output dependent indicator of the integrity SCD of the eroded surface. The given dependences refer to the thickness *H* (30.0 mm, 80.0 mm and 130.0 mm) of high-speed steel EN HS6-5-2C machined with a brass wire electrode AC Brass LP 1000 during a full cut and three other offset cuts.

The following facts can be observed from the reported graphic dependence of the influence of *I* and material thickness *H* in the range of 30.0 mm to 130 mm on SCD with different machining methods. During a full roughing cut with a setting of peak current *I* = 19 A, the value of the SCD eroded surface integrity indicator was recorded in the range of 0.0154 μm·μm^−2^ to 0.0298 μm·μm^−2^, during the first offset cut with a setting of *I* = 14 A in the range 0.0112 μm·μm^−2^ to 0.0240 μm·μm^−2^, at the second offset cut with setting *I* = 8 A in the range of 0.0080 μm·μm^−2^ to 0.0169 μm·μm^−2^ and at the third offset cut with setting *I* = 2 A in the range of 0.0050 μm·μm^−2^ to 0.0097 μm·μm^−2^. In all four cases, the lower value was recorded for material thickness *H* = 130.0 mm.

From the graphical dependence of the influence of *t_on_* and material thickness *H* in the range of 30.0 mm to 130 mm on the SCD with different machining methods, it can be observed that, when the pulse duration was set *t_on_* = 8 μs, the value of the indicator of the integrity of the eroded surface SCD was recorded in the range from 0.0050 μm·μm^−2^ to 0.0179 μm·μm^−2^, at *t_on_* = 16 μs in the range from 0.0062 μm·μm^−2^ to 0.0223 μm·μm^−2^, at *t_on_* = 24 μs in the range from 0.0072 μm·μm^−2^ to 0.0254 μm·μm^−2^ and at *t_on_* = 32 μs in the range from 0.0083 μm·μm^−2^ to 0.0298 μm·μm^−2^. In all four cases, the lower value was recorded for material thickness *H* = 130.0 mm.

From the reported graphic dependence of the influence of *t_off_* and the material thickness *H* in the range of 30.0 mm to 130 mm on the SCD with different machining methods, it can be observed that when the pulse off-time duration *t_off_* = 1 μs was set, the value of the indicator of the integrity of the eroded surface of the SCD was recorded in the range 0.0050 μm·μm^−2^ to 0.0298 μm·μm^−2^, at *t_off_* = 7 μs in the range 0.0062 μm·μm^−2^ to 0.0254 μm·μm^−2^, at *t_on_* = 14 μs in the range 0.0072 μm·μm^−2^ to 0.0223 μm·μm^−2^ and at *t_on_* = 20 μs in the range of 0.0083 μm·μm^−2^ to 0.0179 μm·μm^−2^. Again, the lower value was recorded for material thickness *H* = 130.0 mm.

From the reported graphic dependence of the influence of *U* and material thickness *H* in the range from 30.0 mm to 130 mm *H* on the SCD with different machining methods, it can be observed that, when the voltage of discharge *U* = 60 V was set, the value of the indicator of the integrity of the eroded surface SCD was recorded in the range from 0.0050 μm·μm^−2^ to 0.0240 μm·μm^−2^, at *U* = 70 V in the range 0.0062 μm·μm^−2^ to 0.0219 μm·μm^−2^, at *U* = 80 V in the range 0.0072 μm·μm^−2^ to 0.0298 μm·μm^−2^ and at *U* = 90 V in the range from 0.0083 μm·μm^−2^ to 0.0254 μm·μm^−2^. Again, the lower value was recorded for material thickness *H* = 130.0 mm.

From the reported 2D graphical dependencies in Figure 6, Figure 7, Figure 8 and Figure 9, it can be concluded that the SCD parameter increases significantly with the increasing value of the peak current and the pulse on-time and at the same time with the decreasing value of the pulse off-time and the discharge voltage. This means that the SCD value can be reduced by applying a smaller peak current and pulse on-time duration. At the same time, the SCD value can be reduced by applying a higher value of discharge voltage and a longer pulse off-time. This fact is related to the discharge energy during one discharge cycle, while it is coupled to the MTP setting. A higher peak current produces a greater current density of the discharge channel, through which a large amount of molten material is produced. This subsequently unevenly heats the machined surface, resulting in an uneven distribution of tension in the solidifying layer. The uneven distribution of stress in the solidifying layer is then the carrier of cracks and fissures on the machined surface. At the same time, the higher the value of the peak current applied during WEDM, the more cracks are present on the machined surface, which is reflected in the higher value of the SCD parameter. Cracks occur mainly during the first cooling phase of the machined surface. Cracks may form immediately after it cools down in the second phase. This undesirable effect is also supported by the fact that the long duration of the discharge will allow the transfer of heat to the deeper surface structures of the machined material. This produces a greater thickness of the heat-affected layer, whose significant component is the so-called white layer. The greater thickness of the white layer has a negative impact on the integrity of the eroded surface due to the higher residual stress of the material. The reason is the rapid cooling of the top layer due to the impact of the dielectric liquid, while the inner one solidifies slowly and gradually. As a result, internal stress is concentrated in the subsurface layer, tearing the upper eroded surface.

**Figure 10:**
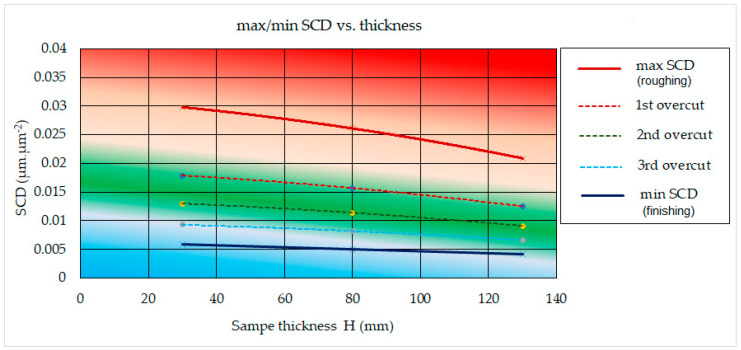
Dependence of min SCD and max SCD on material thickness *H* at WEDM of high-speed steel EN HS6-5-2C with brass wire electrode AC Brass LP 1000.

The graph in Figure 10 shows the dependence of the recorded minimum and maximum values of the surface integrity indicator SCD after WEDM of high-speed steel EN HS6-5-2C with a brass wire electrode AC Brass LP 1000 on the thickness of the processed material *H* in the range from 30.0 mm to 130.0 mm.

From the graphical dependence in Figure 10, a direct relationship between min/max SCD and material thickness *H* can be observed in WEDM of high-speed steel EN HS6-5-2C with brass wire electrode AC Brass LP 1000. At the same time, the biggest difference min/max SCD can be observed at the thickness of the machined material H = 30.0 mm, namely 0.0239 μm·μm^−2^. On the contrary, the smallest difference min/max SCD can be observed at the thickness of the machined material *H* = 130.0 mm, namely 0.0168 μm·μm^−2^. The reason for the lower SCD value with a greater thickness of the machined material *H* is mainly the fact that the heat acting on the machined surface due to electric discharges is more intense and at the same time more evenly spread in all directions and to a greater depth of the base material. As a result, the residual stress in the surface and subsurface layers of the machined material will be reduced, which will also reduce the density of the resulting surface cracks and fissures.

### 3.4. Assessment of the Influence of Selected MTPs on the Microgeometry of the Surface

From the point of view of the validity of the obtained experimental data, a targeted analysis of the influence of selected input factors during electro-erosion machining of high-speed steel EN HS6-5-2C with a brass wire tool electrode AC Brass LP 1000 on the output dependent indicator of the machined surface Ra was performed. The analysis was carried out by the Taguchi method. Based on the experimentally measured data of the roughness parameter of the eroded surface Ra recorded in Table 4, a 4-factor analysis of the influence of selected MTPs during WEDM of high-speed steel EN HS6-5-2C with a brass wire electrode AC Brass LP 1000 was performed using the Taguchi method.

**Figure 11:**
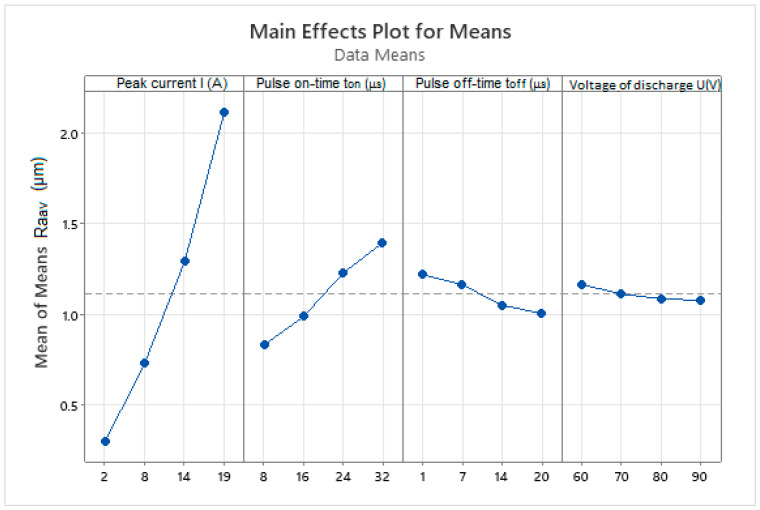
4-factor analysis of the main effect of input MTP on the output qualitative indicator of the machined surface Ra_av_ during WEDM of high-speed steel EN HS6-5-2C.

Based on the average values of the output dependent quality indicator of the machined surface Ra_av_ recorded in Table 4, a 4-factor analysis of the influence of selected MTPs during WEDM of high-speed steel EN HS6-5-2C with brass wire electrode AC Brass LP 1000 was performed using the Taguchi method. The results of the performed 4-factor MTP analysis on 4-levels of their settings and their influence on the output indicator of the roughness of the eroded surface Ra_av_ are presented in the graphs in Figure 11.

From the performed 4-factor analysis of the main influence of the input independent MTP on the output dependent qualitative indicator of the machined surface Ra_av_ during WEDM of high-speed steel EN HS6-5-2C, the following facts were found. Parameter *I* has the greatest influence on the quality indicator of the machined surface Ra_av_, followed by *t_on_*. *U* and *t_off_* have the smallest influence of the assessed parameters. At the same time, it was found that the best quality of the machined surface in terms of the Ra parameter can be achieved with the combination of MTP with the minimum value of the *I* and *t_on_* parameters and at the same time with the maximum value of the *U* and *t_off_* parameters.

**Figure 12:**
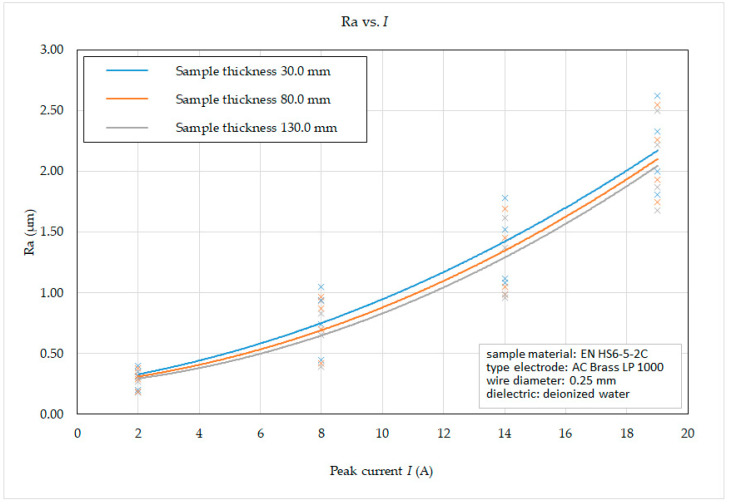
Dependence of Ra on *I* and *H* during WEDM of high-speed steel EN HS6-5-2C.

**Figure 13:**
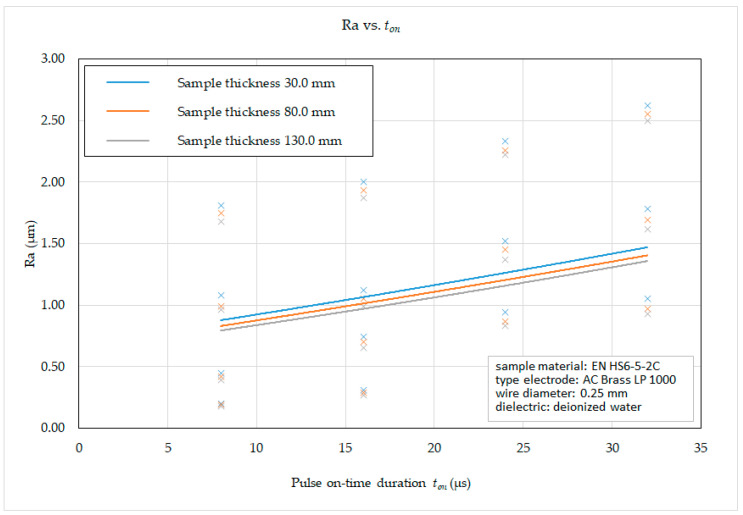
Dependence of Ra on *t_on_* and *H* during WEDM of high-speed steel EN HS6-5-2C.

**Figure 14:**
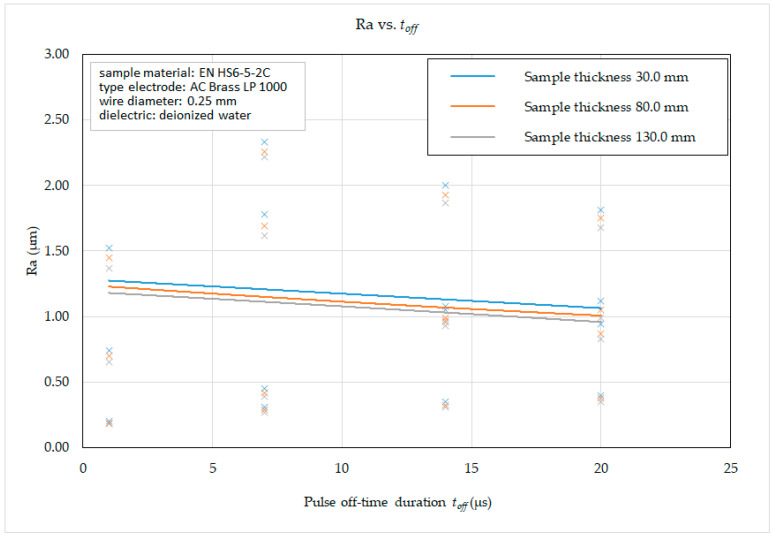
Dependence of Ra on *t_off_* and *H* during WEDM of high-speed steel EN HS6-5-2C.

**Figure 15:**
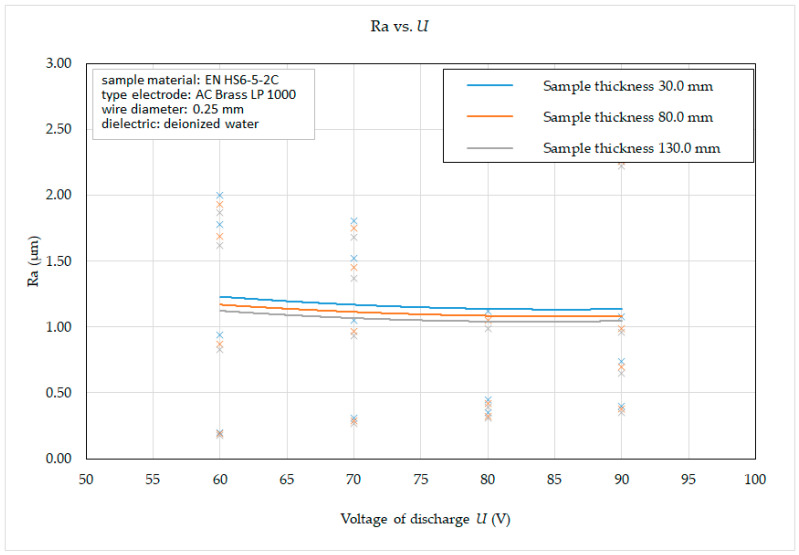
Dependence of Ra on *U* and *H* during WEDM of high-speed steel EN HS6-5-2C.

Subsequently, on the basis of the recorded data, 2D graphic dependencies (Figure 12, Figure 13, Figure 14 and Figure 15) describing the influence of individual input independent MTP on the output dependent indicator of the machined surface Ra were constructed. The given dependencies refer to the thickness *H* (30.0 mm, 80.0 mm and 130.0 mm) of high-speed steel EN HS6-5-2C machined with brass wire electrode AC Brass LP 1000.

From the reported graphical dependence of the roughness parameter of the eroded surface Ra on the peak current *I* and the thickness of the material *H* in the range from 30.0 mm to 130 mm *H* with different machining methods, the following facts can be observed. When setting the peak current *I* = 19 A (full roughing cut), the value of the roughness parameter Ra of the eroded surface was recorded in the range from 1.68 μm to 2.62 μm, at *I* = 14 A (first offset cut) in the range from 0.96 μm to 1.78 μm, at *I* = 8 A (second offset cut) in the range from 0.39 μm to 1.05 μm and at *I* = 2 A (third offset cut) in the range from 0.18 μm to 0.40 μm, while the lower value of Ra was recorded in all cases at material thickness *H* = 130.0 mm. At the same time, it follows from the above that, in the case of WEDM of high-speed steel EN HS6-5-2C with a brass wire electrode AC Brass LP 1000, with an increase in the number of offset cuts, there is a significant decrease in the value of the roughness parameter Ra of the eroded surface.

From the graphical dependence of the roughness parameter of the eroded surface Ra on the pulse on-time duration *t_on_* and the thickness of the material *H* in the range from 30.0 mm to 130 mm with different machining methods, it can be observed that, when the pulse on-time duration *t_on_* = 8 μs was set, the recorded value of the roughness parameter of the eroded surface Ra was in the range from 0.18 μm to 1.81 μm, at *t_on_* = 16 μs in the range from 0.27 μm to 2.0 μm, at *t_on_* = 24 μs in the range from 0.31 μm to 2.33 μm and at *t_on_* = 32 μs in the range from 0.35 μm to 2.62 μm, while the lower values of Ra were recorded in all cases at material thickness *H* = 130.0 mm. At the same time, it follows from the above that, during WEDM of high-speed steel EN HS6-5-2C with a brass wire electrode AC Brass LP 1000, with an increase in the value of the pulse on-time duration *t_on_* parameter, there is a substantial increase in the value of the roughness parameter Ra of the eroded surface.

From the graphical dependence of the roughness parameter of the eroded surface Ra on the pulse off-time duration toff and the thickness of the material *H* in the range from 30.0 mm to 130 mm for different machining methods, it can be observed that, when the pulse off-time duration *t_off_* = 1 μs was set, recorded value of the roughness parameter of the eroded surface Ra w as in the range from 0.18 μm to 2.62 μm, at *t_off_* = 7 μs in the range from 0.27 μm to 2.33 μm, at *t_off_* = 14 μs in the range from 0.31 μm to 2.0 μm and at *t_off_* = 20 μs in the range from 0.35 μm to 1.81 μm, while the lower Ra values were recorded in all cases at the material thickness *H* = 130.0 mm. At the same time, it follows from the above that, during WEDM of high-speed steel EN HS6-5-2C with a brass wire electrode AC Brass LP 1000, with an increase in the value of the pulse off-time duration *t_off_* parameter, there is a slight decrease in the value of the roughness parameter Ra of the eroded surface.

The following facts can be observed from the graphical dependence of the roughness parameter of the eroded surface Ra on the voltage of discharge *U* and the thickness of the material *H* in the range from 30.0 mm to 130 mm with different machining methods. When setting the voltage of discharge *U* = 60 V, the value of the roughness parameter of the eroded surface Ra was recorded in the range from 0.18 μm to 2.0 μm, at *U* = 70 V in the range from 0.27 μm to 1.81 μm, at *U* = 80 V in the range from 0.31 μm to 2.62 μm and at *U* = 90 V in the range from 0.35 μm to 2.33 μm, while the lower Ra values were recorded in all cases at the material thickness *H* = 130.0 mm. At the same time, it follows from the above that, during WEDM of high-speed steel EN HS6-5-2C with brass wire electrode AC Brass LP 1000, the value of the roughness parameter Ra of the eroded surface decreases slightly with the increase in the value of the voltage of discharge parameter *U*.

Based on the obtained 2D graphical dependencies (Figure 12, Figure 13, Figure 14 and Figure 15), it can be concluded that the roughness parameter Ra of the eroded surface increases significantly with the increasing value of peak current *I* and pulse on-time duration *t_on_*. On the other hand, with the increasing value of voltage of discharge *U* and pulse off-time duration *t_off_*, the roughness parameter of the eroded surface Ra improves only to a minimal extent. This means that a better quality of the machined surface for WEDM of high-speed steel EN HS6-5-2C can be achieved by applying a smaller peak current *I* and pulse on-time duration *t_on_* and at the same time a higher value of the parameters voltage of discharge *U* and pulse off-time duration *t_off_*. This is mainly related to the discharge energy during one cycle, where a lower peak current *I* produce lower current density of the discharge channel, the discharge has a shorter time *t_on_*, a longer pulse off-time duration *t_off_* provides more time for the molten surface material to cool, and a higher current has a beneficial effect on removal of particles from the surface of the eroded material. This results in a cleaner and smoother surface.

**Figure 16:**
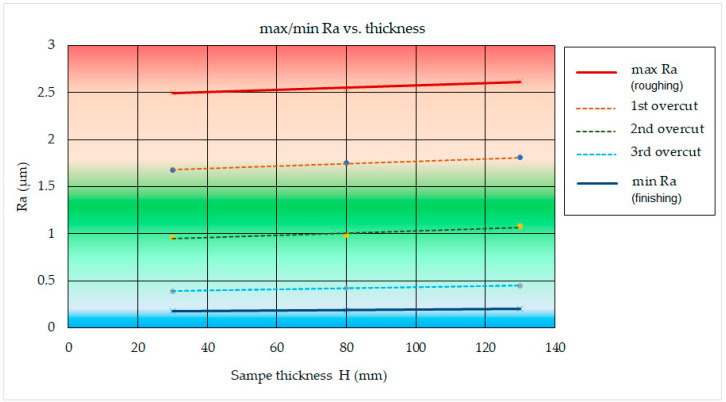
Effect of material thickness *H* on min and max Ra during WEDM of high-speed steel EN HS6-5-2C with brass wire electrode AC Brass LP 1000.

The graph in Figure 16 shows the effect of the thickness of the machined material *H* in the range from 30.0 mm to 130.0 mm on the minimum and maximum value of the roughness parameter of the eroded surface Ra during WEDM of high-speed steel EN HS6-5-2C with a brass wire electrode AC Brass LP 1000 in different cutting mode.

From the graphic dependence (Figure 10), it can be observed that; with the material thickness *H*, the min and max Ra for WEDM of high-speed steel EN HS6-5-2C with brass wire electrode AC Brass LP 1000 change only minimally. This difference ranges from 4.45% to 12.38%. At a constant material thickness due to the application of different discharge energy, a different quality of the machined surface was identified in terms of the Ra parameter. This difference ranges from 2.32 μm to 2.42 μm.

### 3.5. Prediction of SCD and Ra at WEDM for a Broad Range of H by MRM

In order to predict the output qualitative indicators of the machined surface SCD and Ra during WEDM of high-speed steel EN HS6-5-2C with a brass wire electrode AC Brass LP 1000 for a wider spectrum of material thicknesses *H*, Multiple Regression Models (MRM) were compiled at the 95% confidence level. The input parameters of the MRM for the prediction of the output quality parameters SCD and Ra were the input independent variables of the MTP. Specifically, the input parameters peak current *I*, pulse on-time duration *t_on_*, pulse off-time duration *t_off_* and voltage of discharge *U* were included in the MRM. Higher validity of the proposed MRM is represented by the value of determination coefficients (R^2^), which is close to the value of 1. The MRM, which is represented by Equation (2), was compiled on the basis of experimentally measured values with regard to the minimization of the SCD parameter.

(2) $$\begin{array}{l} SCD=0.001276+0.000656\cdot X1+0.000264\cdot X2-0.000063\cdot X3+0.000019\cdot X4+0.000007\cdot {X1}^{2}\\ +0.000001\cdot {X2}^{2}-0.000000\cdot {X4}^{2}+0.000001\cdot X2\cdot X3-0.000002\cdot X3\cdot X4 \end{array}$$

**Figure 17:**
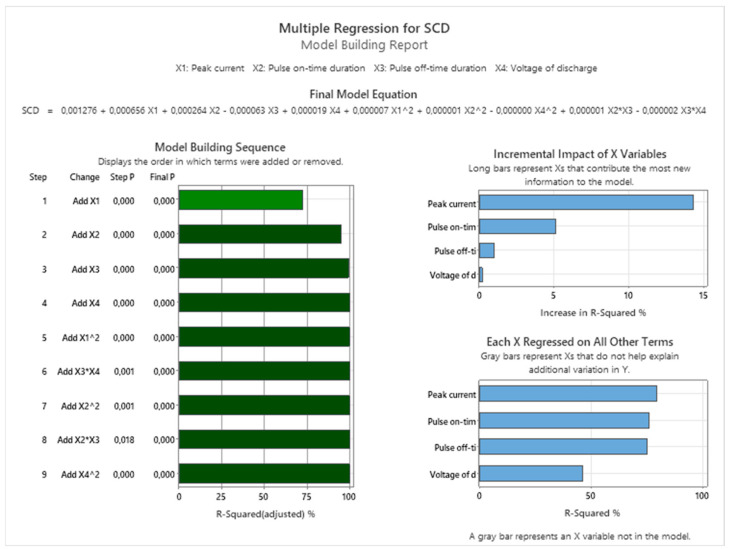
The protocol of the performed Multiple Regression for the output quality parameter SCD in WEDM high-speed steel EN HS6-5-2C.

Figure 17 shows the Multiple Regression for the output qualitative indicator of the machined area of SCD when machining high-speed steel with WEDM technology, including Model Building Sequence and Incremental Impact of Variables. Multiple Regression of the SCD parameter depending on the input MTP was performed with regard to its minimization.

Based on the experimentally measured values, the MRM for the output quality parameter Ra, which is represented by Equation (3), was then compiled:

(3) $$\begin{array}{l} Ra=1.291-0.03667\cdot X1+0.01361\cdot X2+0.05070\cdot X3-0.03338\cdot X4+0.004215\cdot {X1}^{2}\\ +0.000395\cdot {X2}^{2}+0.000207\;\cdot {X4}^{2}+0.000629\;\cdot X1\cdot X4\;-\;0.000394\cdot X2\cdot X3\;-\;0.000700\cdot X3\cdot X4 \end{array}$$

**Figure 18:**
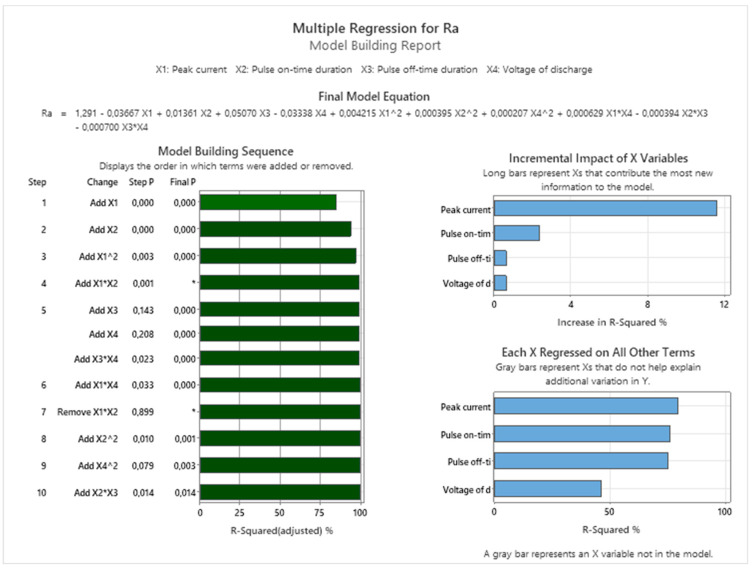
Protocol of the Multiple Regression for the output quality parameter Ra for WEDM of high-speed steel EN HS6-5-2C.

Figure 18 shows the result of the Multiple Regression, which describes the dependence of the output qualitative indicator of the machined area Ra on the input independent MTP for WEDM of high-speed steel EN HS6-5-2C. At the same time, it presents the Model Building Sequence and Incremental Impact of Variables within the Multiple Regression of the Ra parameter with regard to its minimization.

Based on the Multiple Regression, which is described by the created MRM for SCD (2) and Ra (3) in WEDM of high-speed steel EN HS6-5-2C with brass wire electrode AC Brass LP 1000 with regard to their minimization depending on the input MTPs (*I*, *t_on_*, *t_off_* a *U*), the monitored indicators were predicted for a wide range of parameter *H*. The limit of MRM application for machining HSS EN HS6-5-2C by WEDM technology is in the range of material thicknesses from 10 mm to 350 mm. Regression Equation (4) describes the performed prediction of the output quality indicator SCD depending on the parameters Ra and *H*:

(4) SCD=0.011306+0.006612⋅ Ra − 0.000054 ⋅H

**Figure 19:**
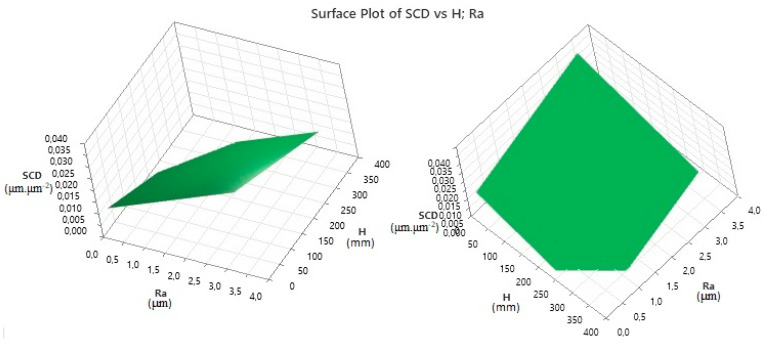
Prediction of the output quality parameter SCD during WEDM of high-speed steel EN HS6-5-2C.

Subsequently, a 3D dependence was drawn (Figure 19), which graphically shows the prediction of the output qualitative indicator of the machined surface SCD during WEDM of high-speed steel EN HS6-5-2C with a brass wire electrode AC Brass LP 1000 in the range of thicknesses *H* = 10.0 mm to 350.0 mm.

From the graphic prediction in Figure 19, it can be observed that, as the thickness *H* of the machined high-speed steel EN HS6-5-2C increases, the predicted value of the output quantitative parameter of the eroded surface SCD also increases. With the thickness of the machined material *H* = 10.0 mm and the roughness of the machined surface Ra = 0.18 μm, the predicted value of the indicator SCD = 0.0073 μm·μm^−2^, and with the roughness Ra = 3.50 μm, the predicted value of the indicator SCD = 0.0392 μm·μm^−2^. With the thickness of the machined material *H* = 350.0 mm and the roughness of the machined surface Ra = 0.18 μm, the predicted value of the indicator SCD = 0.0014 μm·μm^−2^, and with the roughness Ra = 3.50 μm, the predicted value of the indicator SCD = 0.0063 μm·μm^−2^. From the predicted values, it can be concluded that the lowest value of the output quality parameter SCD for WEDM high-speed steel EN HS6-5-2C 2C with brass wire electrode AC Brass LP 1000 can be achieved with the largest thickness of the machined material *H* and at the same time the lowest roughness of the eroded surface Ra.

As in the case of the SCD parameter, the output qualitative indicator Ra was also predicted. This prediction is described by the following Regression Equation (5):

(5) Ra=−1.273+132.04⋅ SCD+0.007192 ⋅H

The limit of MRM application for machining HSS EN HS6-5-2C by WEDM technology with a brass wire electrode AC Brass LP 1000 is in the range of material thicknesses from 10 mm to 350 mm.

**Figure 20:**
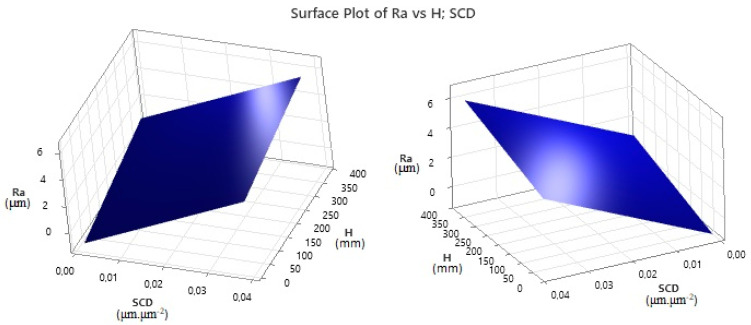
Prediction of the output quality parameter Ra during WEDM of high-speed steel EN HS6-5-2C.

The 3D graphic dependence in Figure 20 shows the prediction of the output quality indicator of the machined surface during WEDM of high-speed steel EN HS6-5-2C with a brass wire electrode AC Brass LP 1000 based on Regression Equation (5) in the thickness range *H* = 10.0 mm up to 350.0 mm.

From the graph in Figure 20, it is evident that, as the thickness *H* of the machined high-speed steel EN HS6-5-2C and the SCD values increase, the predicted value of the output quantitative parameter Ra of the eroded surface increases. With the thickness of the machined material *H* in the range from 10.0 mm to 350 mm and an approximately constant value of the indicator SCD = 0.011 μm·μm^−2^, the predicted roughness of the machined surface Ra increases by approximately 1.96 μm. With a constant thickness of the machined material *H* = 350 mm and a change in the value of the indicator SCD from 0.011 μm·μm^−2^ to 0.04 μm·μm^−2^, the increase in the predicted roughness Ra of the machined surface is approximately 3.83 μm. It follows that, with a change in the value of the SCD parameter, the predicted value of the output quantitative parameter of the eroded surface Ra increases more significantly than with a change in the thickness of the machined material *H*.

Based on the prediction of the values of the output dependent qualitative indicators of the machined area SCD and Ra during WEDM of high-speed steel EN HS6-5-2C with brass wire electrode AC Brass LP 1000 with regard to their minimization in the range of thicknesses *H* = 10.0 mm to 350.0 mm, several facts can be stated. First, it can be stated that the lowest value of both the output dependent quality indicator of the machined area SCD and Ra can be achieved at the lowest value of input independent MTP (*I*, *t_on_*) and at the same time the highest value of MTP (*t_off_*, *U*). At the same time, it can be stated that the SCD and Ra parameters also influence each other. This means that, with a constant thickness *H* of the machined material, when the value of the quality indicator Ra of the machined surface decreases, the value of the SCD indicator also decreases. At the same time, its lowest predicted value of 0.0014 μm·μm^−2^ can be achieved with the thickness of the machined material *H* = 350.0 mm and the roughness of the eroded surface Ra = 0.18 μm. On the contrary, the highest predicted value of the indicator SCD = 0.0392 μm·μm^−2^ can be achieved with the thickness of the machined material *H* = 10.0 mm and the roughness of the eroded surface Ra = 3.50 μm.

Based on the research performed in the field of machined surface integrity in WEDM of high-speed steels, the following general conclusions can be drawn in relation to the qualitative indicator SCD of the eroded surface. The magnitude of the SCD indicator is largely influenced by MTP, which is consistent with the results of several authors. The greatest influence is for *I* and *t_on_*. On the contrary, the smallest impact is for *t_off_* and *U*. Furthermore, it can be stated that the minimum value of the SCD indicator corresponds to the minimum value of the parameters *I*, *t_on_* and at the same time the maximum value of the parameters *t_off_* and *U*. However, this already contradicts the claim of Wang et al. [65]. Their research showed that the greatest impact on minimizing SCD shows high values of *I* and *t_off_*. Partially contradictory results were also obtained by Shather et al. [66], who claim that the smallest disintegrity of the eroded surface can be achieved with a low value of the parameter *I* and at the same time a high value of the parameters *t_on_* and *t_off_*. Consistent with our findings were the results obtained by Rahimi et al. [66]. However, they only took into account the parameters *I* and *t_on_* in the experiments performed in relation to SCD. Nevertheless, it should be stressed here that in neither of these cases were the experimental conditions identical.

## 4. Conclusions

The experimental research was focused on identifying two important qualitative indicators of the eroded surface during WEDM of high-speed steel EN HS6-5-2C with a brass wire electrode labelled AC Brass LP 1000. The qualitative indicators assessed were SCD and Ra. Their values were examined in relation to MTP (*I*, *t_on_*, *t_off_* a *U*) at four levels of settings and in the entire cross-section of the eroded surface of samples with thicknesses *H* = 30.0 mm, 80.0 mm and 130 mm. In accordance with the Taguchi method, 16 (L1 to L16) experimental specimens were made for each material thickness with different combinations of MTP settings, corresponding to full cut conditions and three offset cuts. Based on the experimental measurements, the output monitored indicator of the roughness of the eroded surface Ra in the range from 0.18 μm to 2.62 μm and the indicator of the surface integrity SCD in the range from 0.0041 μm to 0.0298 μm were identified. The factor analysis revealed that the parameters *I* and *t_on_* have the greatest influence on the given output dependent indicators of the eroded area. On the contrary, the parameters *t_off_* and *U* have the least influence. The results of experimental measurements also showed that lower SCD values at identical MTP settings were recorded when machining thicker materials. The difference between the SCD values at 30.0 mm and 130.0 mm thickness was approximately 30%. This means that, in addition to the MTP, the thickness of the machined material also has a significant impact on the SCD indicator. The difference in the measured values of the output qualitative indicator Ra of the eroded surface within the performed experiment ranged from 4.45% to 12.38%. In addition, a decrease in the SCD indicator value was identified with an increasing number of offset cuts. Subsequently, based on the obtained and numerically processed results, MRMs were compiled at a probability level of 95%, which allow the prediction of given output quality indicators of the eroded surface during WEDM of high-speed steel EN HS6-5-2C in the thickness range from 10.0 mm to 350.0 mm. By applying the obtained MRMs, the SCD value was predicted for the given extent in the range from 0.0014 μm to 0.0392 μm and the corresponding roughness Ra of the eroded surface in the range from 0.18 μm to 3.50 μm. The performed prediction was visualized through 3D plots.

## Figures and Tables

**Table 1:** Properties of HSS EN HS6-5-2C.

**Physical Properties**				
Thermal Conductivity at 20 °C (W·m^−1^·K^−1^)	Specific Electric resist. (Ω·mm^2^·m^−1^)	Melting Point °C	Specific heat (J·kg^−1^·K^−1^)	Density (kg·dm^−3^)
24	0.54	4680	460	8.12
**Mechanical Properties**				
Compression strength limit MPa	Modulus of elasticity 10^3^ N·m^−2^	Poisson’s ratio	Machinability (1% carbon steel)	Hardness HRC
3250	217	0.30	50.0%	65

**Table 2:** The main technical parameters of the machine AgieCharmilles CUT E 350.

Main Specifications	Value
Travel X/Y/Z	350 × 250 × 250 mm
Max. taper angle/height	±30/50° mm
Max. workpiece dimensions	820 × 680 × 250 mm
Wire diameter range	0.10–0.30
Dimensions of complete equipment	1850 × 3050 × 2220 mm
Wire feed rate	3000 mm·min^−1^
Min. surface roughness Ra	0.14 μm
Max. workpiece weight	400 kg
Max. wire spool weight	8 kg

**Table 3:** SCD results according to the 4-factor 4-level design of experiments.

Run No.	MTP				Experimental Results								
	*I* (I)	*t_on_* (μs)	*t_off_* (μs)	*U* (V)	Average Measured Value SCD (μm·μm^−2^) for Material Thickness *H* (mm)			Standard Deviation of Measured Value SCD for Material Thickness *H* (mm)			Calculated Value for Range of Material Thickness *H* = 30.0 mm to 130.0 mm		Percentage (%)
					30.0	80.0	130.0	30.0	80.0	130.0	SCD_av_ (μm·μm^−2^)	Standard Deviation	
1	2	8	1	60	0.0059	0.0051	0.0041	0.00016	0.00012	0.00008	0.0050	0.00074	30.51
2	2	16	7	70	0.0072	0.0063	0.0051	0.00019	0.00016	0.00012	0.0062	0.00089	29.95
3	2	24	14	80	0.0084	0.0073	0.0059	0.00021	0.00012	0.00016	0.0072	0.00104	30.04
4	2	32	20	90	0.0097	0.0085	0.0068	0.00016	0.00021	0.00012	0.0083	0.00120	30.24
5	8	8	7	80	0.0093	0.0082	0.0066	0.00012	0.00014	0.00016	0.0080	0.00112	29.29
6	8	16	1	90	0.0130	0.0114	0.0091	0.00008	0.00021	0.00021	0.0112	0.00162	30.26
7	8	24	20	60	0.0133	0.0116	0.0093	0.00021	0.00016	0.00016	0.0114	0.00165	30.25
8	8	32	14	70	0.0169	0.0148	0.0119	0.00016	0.00019	0.00016	0.0145	0.00205	29.59
9	14	8	14	90	0.0130	0.0114	0.0091	0.00012	0.00016	0.00012	0.0112	0.00160	29.92
10	14	16	20	80	0.0151	0.0132	0.0105	0.00008	0.00021	0.00021	0.0129	0.00190	30.68
11	14	24	1	70	0.0219	0.0192	0.0154	0.00016	0.00008	0.00021	0.0188	0.00265	29.53
12	14	32	7	60	0.0240	0.0210	0.0168	0.00016	0.00012	0.00012	0.0206	0.00297	30.14
13	19	8	20	70	0.0179	0.0157	0.0125	0.00021	0.00016	0.00016	0.0154	0.00223	30.30
14	19	16	14	60	0.0223	0.0195	0.0156	0.00012	0.00021	0.00016	0.0191	0.00273	29.94
15	19	24	7	90	0.0254	0.0223	0.0178	0.00021	0.00016	0.00012	0.0218	0.00309	29.70
16	19	32	1	80	0.0298	0.0261	0.0209	0.00012	0.00012	0.00016	0.0256	0.00074	29.79

**Table 4:** Ra results according to the 4-factor 4-level design of experiments.

Run No.	MTP				Experimental Results								
	*I* (I)	*t_on_* (μs)	*t_off_* (μs)	*U* (V)	Average Measured Value Ra (μm) for Material Thickness *H* (mm)			Standard Deviation of Measured Value Ra for Material Thickness *H* (mm)			Calculated Value for Range of Material Thickness *H* = 30.0 mm to 130.0 mm		Percentage (%)
					30.0	80.0	130.0	30.0	80.0	130.0	Ra_av_ (μm)	Standard Deviation	
1	2	8	1	60	0.20	0.19	0.18	0.0047	0.0047	0.0082	0.19	0.0096	11.48
2	2	16	7	70	0.31	0.29	0.27	0.0082	0.0082	0.0047	0.29	0.0150	11.83
3	2	24	14	80	0.35	0.32	0.31	0.0082	0.0047	0.0047	0.33	0.0178	12.38
4	2	32	20	90	0.40	0.38	0.35	0.0082	0.0082	0.0047	0.38	0.0191	11.67
5	8	8	7	80	0.45	0.42	0.39	0.0047	0.0047	0.0047	0.42	0.0218	11.94
6	8	16	1	90	0.74	0.70	0.65	0.0047	0.0125	0.0047	0.70	0.0340	11.31
7	8	24	20	60	0.94	0.87	0.83	0.0094	0.0047	0.0094	0.88	0.0455	11.74
8	8	32	14	70	1.05	0.97	0.93	0.0082	0.0082	0.0125	0.98	0.0511	11.75
9	14	8	14	90	1.08	0.99	0.96	0.0047	0.0047	0.0082	1.01	0.0530	11.38
10	14	16	20	80	1.12	1.05	0.99	0.0125	0.0082	0.0047	1.05	0.0504	11.04
11	14	24	1	70	1.52	1.45	1.37	0.0082	0.0094	0.0094	1.44	0.0626	10.09
12	14	32	7	60	1.78	1.69	1.62	0.0047	0.0125	0.0082	1.70	0.0640	8.82
13	19	8	20	70	1.81	1.75	1.68	0.0082	0.0047	0.0094	1.74	0.0545	7.37
14	19	16	14	60	2.00	1.93	1.87	0.0047	0.0082	0.0082	1.93	0.0517	6.34
15	19	24	7	90	2.33	2.26	2.22	0.0163	0.0125	0.0125	2.27	0.0465	4.86
16	19	32	1	80	2.62	2.55	2.50	0.0082	0.0082	0.0047	2.56	0.0479	4.45

## Data Availability

All data are published with the paper.

## Nomenclature

A	area (µm^2^)
*H*	thickness (mm)
HSS	high speed steels
*I*	peak current (A)
LC	crack length (µm)
MRM	multiple regression models
MTP	main technological parameters
Ra	parameter of surface roughness (µm)
SCD	surface crack density μm·μm^−2^
SDM	scanning digital microscope
*t_on_*	pulse on-time duration (µs)
*t_off_*	pulse off-time duration (µs)
*U*	voltage of discharge (V)
WEDM	wire electrical discharge machining
